# Knowledge, attitudes and sexual behavior concerning AIDS among college students in Guangzhou, China: a cross-sectional questionnaire survey

**DOI:** 10.3389/fpubh.2025.1595827

**Published:** 2025-05-20

**Authors:** Yanjun Yang, Shaomin Wu, Yuan Tang

**Affiliations:** ^1^Panyu District Center for Disease Prevention and Control, Guangzhou, China; ^2^Department of Medical Statistics, School of Public Health, Sun Yat-sen University, Guangzhou, China; ^3^Nansha District People’s Hospital, Guangzhou, China

**Keywords:** aids, knowledge, attitudes, sexual behavior, college students, questionnaire

## Abstract

**Background:**

Despite global efforts to control human immunodeficiency virus (HIV) among adolescents, the number of new infections among adolescents continues to increase. The increasingly widespread HIV epidemic among Chinese college students indicates an urgent need for more effective services in this context. To meet this need, we conducted a survey that aimed to produce a clear understanding of knowledge and sexual behavior concerning acquired immunodeficiency syndrome (AIDS) among college students. This study can serve as a reference for policy-makers and university administrators seeking to implement more targeted measures in this context.

**Methods:**

In November 2024, a cross-sectional internet questionnaire survey was distributed at 13 universities in Guangzhou, China. The chi-square test was performed to examine the differences among respondents who exhibited different characteristics. A multivariate logistic regression analysis was conducted to explore the main influences on college students’ AIDS knowledge. Confidence intervals that did not contain zero or *p* values < 0.05 were considered to indicate statistical significance.

**Results:**

A total of 12,632 valid questionnaires were collected. On this basis, a total of 11,587 (91.73%) students were determined to possess AIDS knowledge. The main influences on college students’ AIDS knowledge were age, school classification, major, accommodation method, place of origin and average monthly living expenses. The proportion of students who reported a history of sexual behavior increased alongside students’ grade. The relevant values were as follows: freshmen (5.16%), sophomores (11.9%), fourth-year and fifth-year students (15.59%), master’s students (29.27%) and doctoral students (55.22%). A total of 6.63% of the respondents who had engaged in sexual behaviors reported that they had engaged in noncommercial sex with causal sexual partners. During the past year, the percentages of respondents who did not insist on using condoms during sexual activities with their casual sexual partners, male same-sex sexual partners, or commercial sexual partners were 30.58, 51.81, and 81.25%, respectively. The percentages of students who possessed AIDS knowledge and insisted on using condoms during sexual activities with “casual sexual partners (72.16%)” or “male same-sex sexual partners (48.19%)” were greater than the corresponding percentages of students who did not possess such knowledge (*p* < 0.01). Individuals who did not possess AIDS knowledge reported that they did not insist on using condoms during sexual activities with male same-sex sexual partners. “Did not buy a condom” was identified as the main reason for the failure to use condoms during sexual activities with casual sexual partners (26.43%) and commercial sexual partners on the basis of monetary transactions (48.00%). The main reason for failing to use condoms during same-sex sexual activities was “I did not think that it was necessary to use it” (41.67%). The percentage of college students included in the survey who reported that they had acquired AIDS knowledge from social software was the highest (76.96%). A total of 30.90% of the respondents reported that their favorite way of acquiring AIDS knowledge was through their school courses.

**Conclusion:**

The level of AIDS knowledge exhibited by college students is affected by various factors. Improvements in AIDS knowledge can help raise awareness of the need for self-protection during high-risk sexual activities among college students. The risk of contracting AIDS and other sexually transmitted diseases can be reduced through the use of condoms. Social networks are the main source by which college students acquire AIDS knowledge, although such students typically hope to acquire AIDS knowledge from their school courses.

## Introduction

Acquired immunodeficiency syndrome (AIDS) has become a major public health problem at the global level (1, 2). In 2023, an estimated 39.9 million patients had been infected with human immunodeficiency virus (HIV) worldwide (3). Despite the impressive progress that has been made in efforts to reduce rates of AIDS in recent years, global initiatives have called for the number of new HIV infections to be controlled at 370,000 by 2025 and 335,000 by 2030. The most up-to-date estimates indicate that although the world is progressing in the correct direction, countries worldwide remain far from achieving these goals (4). In 2023, approximately 1 million adolescents between the ages of 15 and 19 were infected with HIV worldwide. Adolescents account for approximately 3% of all HIV infections and approximately 12% of new HIV infections among adults. Aside from Sub-Saharan Africa, Asia and Latin America contain the highest number of HIV-positive adolescents (5). Adolescents and young adults account for an increasing share of the global HIV burden (6). As a sexually active population, young students constitute a key group for AIDS prevention and control efforts in China (7). In recent years, approximately 3,000 cases of HIV infection among young students have been reported in China each year, and sexual activity has been identified as the main route of transmission; furthermore, efforts targeting epidemic prevention and control among young students have encountered severe challenges (8, 9).

The statistics provided by the monitoring report management module of the China Disease Prevention and Control Information System indicated that in December 31, 2023, more than 16,000 living patients with HIV/AIDS resided in Guangzhou, China, and adolescents between the ages of 15 and 24 years accounted for 20.9–23.5% of such cases. From 2018 to 2023, adolescents between the ages of 15 and 24 years in Guangzhou accounted for 3.4–5.0% of new reports of HIV/AIDS. Among newly infected patients in 2023, individuals who had obtained a college degree or higher level of accounted for 51.8% of the total. Highly educated men who have sex with men are a high-risk group with respect to HIV infection and transmission in Guangzhou. To our knowledge, no surveys have yet been conducted to measure AIDS knowledge, sexual attitudes, and behaviors among Chinese college students simultaneously. Therefore, this study aimed to describe the sexual attitudes, sexual behavior, AIDS knowledge, and use of preventative services exhibited by college students in Guangzhou, China.

## Materials and methodology

### Data source

In November 2024, a cross-sectional survey was conducted at 13 universities in Guangzhou, China. Respondents used their mobile phones to scan a quick response (QR) code that enabled them to log into the Wenjuanxing survey platform^1^ to facilitate data collection. A stratified cluster random sampling method was used to select 13 universities from all universities in Guangzhou; the sample included three comprehensive universities, three medical universities, three science and technology universities, two art universities, one normal university, and one language university. In the selected schools, undergraduate students in each grade from no less than two classes were randomly screened, as were graduate students at each school from no less than two classes. All responses to the questionnaire were provided anonymously by the respondents, and the information collected as part of this survey was provided voluntarily. Questionnaires were linked with respondents’ mobile phone numbers, and each phone number was limited to one questionnaire. A total of 12,662 questionnaires were collected. On this basis, 30 questionnaires that were completed in less than 120 s or that featured the same option as a selection for multiple questions were excluded. The remaining 12,632 valid questionnaires were included in the analysis, for an effective rate of 99.76%.

### Survey design

In the first part of the study, the sociodemographic characteristics of the respondents were collected, including their gender, age, nationality, school classification, major, grade, accommodation methods, family residence, places of origin, and average monthly living expenses.

In the second part of the study, we used the “Questionnaire Concerning AIDS Knowledge among Young Students,” which was compiled by the Chinese Center for Disease Control and Prevention to evaluate the respondents’ levels of HIV-related knowledge (10). The questionnaire contained 8 questions. Scores on the questionnaire were calculated as follows: each correct answer was awarded 1 point, and the total score represented the total number of correct answers (0–8 points). A score of 6 or higher was considered to indicate the presence of such knowledge.

In the third section, the respondents’ sexual attitudes, sexual behaviors, and health education pertaining to AIDS were evaluated.

### Statistical methods

Mplus 8.3 software was used to conduct a confirmatory factor analysis (CFA) with the goal of testing the construct validity of the Questionnaire Concerning AIDS Knowledge among Young Students. The parameters were estimated via the weighted least square mean and variance adjusted (WLSMV) method. These rates, percentages and the corresponding 95% confidence intervals (CIs) were used to describe the demographic variables, i.e., AIDS knowledge, awareness, sexual attitudes and behaviors, and the use of preventative services. Data analysis was performed with the assistance of SPSS 25.0 software. Percentages and 95% CIs were used to describe categorical variables. The chi-square test was performed to examine the differences between two or more categorical variables. Spearman’s correlation was used to evaluate the correlations among major statistical variables. A multivariate logistic regression analysis was conducted to explore the main influences on college students’ knowledge of AIDS. All tests performed as part of this research were two-sided, and the level of significance was set at *p* < 0.05.

## Results

### Sociodemographic characteristics

A total of 12,632 valid questionnaires were included in the analysis, including 5,973 (47.28%) responses provided by males and 6,659 (52.72%) provided by females. The respondents were mainly between the ages of 18 and 22 years; this age group accounted for 85.70% of the total. The respondents were recruited from six types of universities, including comprehensive universities (21.41%), medical universities (37.69%), science and technology universities (21.82%), art universities (10.32%), normal universities (4.91%), and language universities (3.86%) (see Table 1).

**Table 1:** General demographic characteristics (*n* = 12,632).

Item	Category	Number	Percentage (%)
Sex	Male	5,973	47.28
	Female	6,659	52.72
Age (years)	<18	236	1.87
	18–	3,459	27.38
	19–	2,703	21.40
	20–	1,993	15.78
	21–	1,529	12.10
	22–	1,141	9.03
	≥23	1,571	12.44
Nationality	Han	12,026	95.20
	Minority	606	4.80
School type	Comprehensive University	2,704	21.41
	Medical University	4,761	37.69
	Polytechnic and Technological University	2,756	21.82
	Art University	1,303	10.32
	Normal University	620	4.91
	Language University	488	3.86
Specialty	Engineering	4,408	34.90
	Medicine	2,904	22.99
	Science	1,799	14.24
	Management	1,135	8.99
	Literature	879	6.96
	Economics	518	4.10
	Jurisprudence	369	2.92
	Pedagogy	386	3.06
	Others (philosophy, history, military science, agriculture, etc.)	234	1.85
Current stage of study	Freshman	4,940	39.11
	Sophomore	2,246	17.78
	Junior	1,937	15.33
	Senior	1,629	12.90
	Postgraduate	1,650	13.06
	Doctoral	230	1.82
Lodging	Student dormitories	12,230	96.82
	Living with family/shared flats/living alone	402	3.18
Registered permanent residence	City	8,070	63.89
	Township/Urban–rural junction	2,598	20.57
	Countryside	1,964	15.55
Family status	Family of origin	11,593	91.77
	Combined families	339	2.68
	Divorced families	582	4.61
	Others	118	0.93
Region of source of students	Chinese mainland	12,187	96.48
	Hong Kong/Macau/Taiwan	445	3.52
Average monthly living expenses (yuan)	<1,000	574	4.54
	1,000–	7,390	58.50
	2,000–	3,862	30.57
	>3,000	806	6.38

### Reliability and validity tests

**Figure 1:**
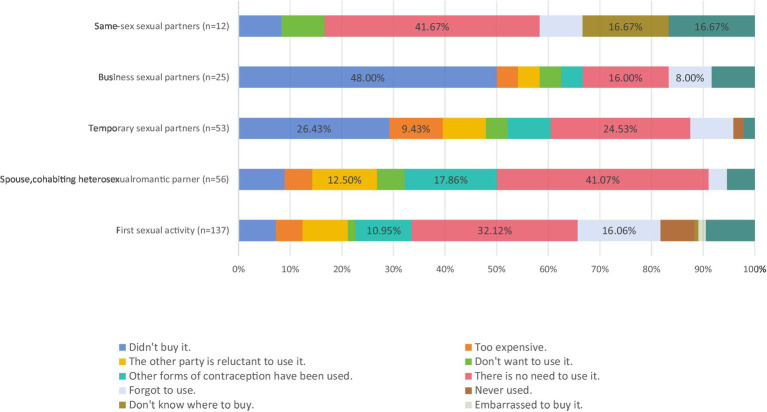
The main reasons for not using condoms.

The reliability and validity of the AIDS-related measurements were tested. Internal consistency reliability, convergent validity and construct validity were used to test the reliability and validity of the module used to investigate respondents’ levels of AIDS knowledge. The Cronbach’s α coefficient was calculated with the assistance of SPSS 25.0 to test the internal consistency reliability of the questionnaire. A CFA was conducted to check the construct validity with the assistance of Mplus 8.4. The Kaiser-Meyer-Olkin (KMO) value for this scale was 0.762, which was statistically significant according to Bartlett’s test (*p* < 0.001). The results of the CFA indicated that the comparative fit index (CFI) was 0.459, the Tucker–Lewis index (TLI) was 0.278, the standardized root mean square residual (SRMR) was 0.204, and the root mean square error of approximation (RMSEA) was <0.001. All factor loadings were >0.5. The Cronbach’s α coefficient for this scale was 0.685. These results revealed that this scale exhibited good reliability and validity (Figure 1).

### AIDS knowledge

A total of 11,587 (91.73, 95% CIs: 91.25–92.21%) students were determined to possess AIDS knowledge, 5,665 of whom (accounting for 44.85% of the total, 95% CIs: 43.98–45.72%) answered all the items included in the questionnaire correctly. In this context, the item “After engaging in high-risk behaviors (such as sharing needles and syringes/unsafe sexual behaviors), you should actively seek HIV testing and counseling” was associated with the highest accuracy rate (97.97, 95% CIs: 97.72–98.21%). The item “The HIV epidemic among students has increased; the main mode of transmission is male homosexuality, followed by heterosexuality” was associated with the lowest accuracy rate (80.84, 95% CIs: 80.16–81.53%) (see Table 2).

**Table 2:** Cognitive levels on the HIV/AIDS knowledge among the college students in Guangzhou, China (*n* = 12,632).

Question	Correct	Incorrect	Have no idea	Awareness percentage (95%CI)
q1: AIDS a serious and incurable infectious disease	10,341	1,921	370	81.86 (81.19–82.54)
q2: At present, the prevalence of AIDS among young students in China is growing rapidly, and the main mode of transmission is male homosexual sex, followed by heterosexual sex	10,212	1,483	937	80.84 (80.16–81.53)
q3: It is impossible to judge whether a person is infected with HIV by their appearance	10,967	928	737	86.82 (86.23–87.41)
q4: Ordinary life and study contact do not infect HIV	11,154	958	520	88.30 (87.74–88.86)
q5: Consistent and correct use of condoms can reduce the risk of contracting and transmitting HIV	12,094	257	281	95.74 (95.39–96.09)
q6: The use of new drugs (e.g., methamphetamine, ecstasy, K powder, etc.) increases the risk of HIV infection	10,964	889	779	86.80 (86.21–87.39)
q7: HIV testing and counseling should be sought after high-risk behaviors (e.g., needle sharing, drug use/unsafe sex, etc.)	12,375	71	186	97.97 (97.72–98.21)
q8: The rights and interests of HIV-infected persons such as marriage, employment, and school enrollment are protected by Chinese laws	10,842	430	1,360	85.83 (85.22–86.44)

### Influences on AIDS knowledge

The results of a chi-square analysis revealed that students aged ≥20 years (χ^2^ = 5.324, *p* < 0.05), graduate students (χ^2^ = 5.324, *p* < 0.05), undergraduate students in years 3–5 (χ^2^ = 5.090, *p* < 0.05), students living in dormitories (χ^2^ = 16.009, *p* < 0.001), those whose place of origin was mainland China (χ^2^ = 168.937, *p* < 0.001), and those whose average monthly living expenses were ≥1,000 RMB exhibited higher levels of AIDS knowledge. The levels of knowledge exhibited by college students in different schools differed (χ^2^ = 108.121, *p* < 0.001); namely, the level of knowledge exhibited by students at science and technology universities was the highest (94.45%), whereas that exhibited by students at art universities was the lowest (85.34%). The levels of knowledge exhibited by college students with different majors also differed (χ^2^ = 198.180, *p* < 0.001); namely, the level of knowledge exhibited by college students majoring in engineering was the highest (94.06%), whereas that exhibited by college students majoring in philosophy, history, military affairs, or agronomy was the lowest (79.91%) (see Table 3).

**Table 3:** Knowledge of HIV/AIDS (*n* = 12,632).

Item		Number	Aware	Percentage (%)	χ^2^	*P*
Sex	Male	5,973	5,474	91.65	0.099	0.752
	Female	6,659	6,113	91.80		
Age(years)	<20	6,398	5,833	91.17	5.324	0.021
	≥20	6,234	5,754	92.30		
School type	Polytechnic and Technological University	2,756	2,603	94.45	108.121	<0.001
	Normal University	620	583	94.03		
	Language University	488	458	93.85		
	Medical University	4,761	4,378	91.96		
	Comprehensive University	2,704	2,453	90.72		
	Art University	1,303	1,112	85.34		
Specialty	Engineering	4,408	4,146	94.06	198.180	<0.001
	Jurisprudence	369	345	93.50		
	Science	1,799	1,668	92.72		
	Medicine	2,904	2,677	92.18		
	Management	1,135	1,036	91.28		
	Literature	879	779	88.62		
	Pedagogy	386	335	86.79		
	Economics	518	414	79.92		
	Others (philosophy, history, military science, agriculture, etc.)	234	187	79.91		
Current stage of study	Undergraduate	10,752	9,833	91.45	7.179	0.007
	Postgraduate/doctoral	1,880	1,754	93.30		
Grade	Freshman/sophomore	7,186	6,541	91.02	5.090	0.024
	Junior/senior	3,566	3,292	93.30		
Lodging	Student dormitories	12,230	11,240	97.91	16.009	<0.001
	(Living with family/shared flats/living alone)	402	347	86.32		
Registered permanent residence	Chinese mainland	12,187	11,253	92.34	168.937	<0.001
	Hong Kong/Macau/ Taiwan	445	334	75.06		
Monthly expenses	Under 1,000 yuan	574	500	87.11	16.909	<0.001
	1,000 yuan and above	12,058	11,087	91.95		

The correlation coefficients among variables were calculated before the multivariate analysis was conducted. Strong correlations were observed among the variables pertaining to students’ age, school classification, major, academic level, and place of origin (see Table 4).

**Table 4:** Spearman correlation analysis among the main factor.

	Age (years)	School type	Specialty	Lodging	Region of source of students	Monthly expenses
Age(years)	1	−0.055**	−0.032**	−0.077**	0.052**	−0.029**
School type	−0.055**	1	0.255**	0.079**	0.195**	0.017
Specialty	−0.032**	0.255**	1	0.083**	0.29**	0.024**
Lodging	0.077**	0.079**	0.083**	1	0.049**	0.002
Region of source of students	0.052**	0.195**	0.290**	0.049**	1	0.004
Monthly expenses	−0.029**	0.017	−0.024**	0.002	0.004	1

**P < 0.01.

The results of a multivariate logistic regression analysis revealed that the main influences on college students’ levels of AIDS knowledge were age, school classification, majors, accommodation method, place of origin, and average monthly living expenses. In this context, the level of AIDS knowledge possessed by college students who were 20 years old or older was 1.152 times that possessed by students who were younger than 20 years old. The level of AIDS knowledge possessed by students from mainland China was 2.555 times that possessed by students from Hong Kong, Macau, and Taiwan. Finally, the level of AIDS knowledge possessed by students whose average monthly living expenses were greater than 1,000 RMB was 1.695 times that possessed by other students (see Table 5).

**Table 5:** Multivariate unconditional logistic regression on awareness of HIV/AIDS.

Variables	*B*	*S.E.*	*Wald x* ^2^	*P*	*OR*	95%*CI*
Constant	−3.034	0.086	1250.406	<0.001		
Age						
≥20 years old	0.141	0.067	4.409	0.036	1.152	1.0096–1.314
<20 years old (Ref.)						
School type						
Comprehensive University	0.245	0.107	5.224	0.022	1.278	1.036–1.577
Medical University	0.444	0.089	24.662	<0.001	1.558	1.308–1.857
Others (Language University, Normal University, Polytechnic, Technological University, etc.)	0.729	0.135	29.314	<0.001	2.073	1.592–2.700
Art University (Ref.)						
Specialty						
Economics	0.284	0.116	6.044	0.014	1.329	1.059–1.667
Pedagogy/literature	0.334	0.129	6.760	0.009	1.397	1.086–1.798
Management	0.720	0.174	17.123	<0.001	2.054	1.461–2.889
Medicine/science/jurisprudence/engineering	0.770	0.196	15.411	<0.001	2.159	1.470–3.171
Others (philosophy, history, military science, agriculture, etc.) (Ref.)						
Lodging						
Student dormitories	0.348	0.155	5.038	0.025	1.417	1.045–1.921
(Living with family/Shared flats/Living alone) (Ref.)						
Registered permanent residence						
Chinese mainland	0.938	0.177	28.009	<0.001	2.555	1.805–3.617
Hong Kong, Macau or Taiwan (Ref.)						
Monthly expenses (yuan)						
≥1,000 yuan	0.528	0.132	15.999	<0.001	1.695	1.309–2.195
<1,000 yuan (Ref.)						

### Sexual attitudes

A total of 6,176 (48.89, 95% CIs: 48.02–49.76%) students reported that they accepted sexual behavior among college students. Among males and females, these percentages were 63.65% (95% CIs: 62.43–64.87%) and 35.65% (95% CIs: 34.50–36.80%), respectively, and this difference was statistically significant (*χ*^2^ = 5.344, *p =* 0.021).

Among college students who reported their acceptance of such sexual behavior, the percentage of students who possessed AIDS knowledge (49.23%) was higher than that of students who did not possess AIDS knowledge (45.17%), and this difference was statistically significant (χ^2^ = 6.324, *p* = 0.012). In this context, the percentage of males who possessed AIDS knowledge (64.10%) was higher than that of males who did not possess AIDS knowledge (59.00%), and this difference was also statistically significant (χ^2^ = 5.344, *p =* 0.021). Furthermore, the percentage of females who possessed AIDS knowledge (64.10%) was higher than that of females who did not possess AIDS knowledge (59.00%), and this difference was statistically significant (χ^2^ = 4.561, *p =* 0.033). Finally, the percentage of graduate students (including both master’s and doctoral students) who possessed AIDS knowledge (65.79%) was higher than that of those who did not possess AIDS knowledge (48.41%), and this difference too was statistically significant (χ^2^ = 15.533, *p <* 0.001) (see Table 6).

**Table 6:** Attitudes toward sexual behavior among the college students in Guangzhou China (*n* = 12,632).

Item		Knowledge of HIV/AIDS	Number	Accept college student sexual behavior		χ^2^	*P*
				Number	%		
Gender	Male	Unaware	522	308	59.00	5.344	0.021
		Aware	5,451	3,494	64.10		
		Total	5,973	3,802	63.65		
	Female	Unaware	523	164	31.36	4.561	0.033
		Aware	6,136	2,210	36.02		
		Total	6,659	2,374	35.65		
Grade	Undergraduate	Unaware	919	411	44.72	0.813	0.367
		Aware	9,833	4,550	46.27		
		Total	10,752	4,961	46.14		
	Freshman	Unaware	435	179	41.15	0.580	0.446
		Aware	4,505	1,939	43.04		
		Total	4,940	2,118	42.87		
	Sophomore	Unaware	210	107	50.95	1.132	0.287
		Aware	2,036	959	47.10		
		Total	2,246	1,066	47.46		
	Junior	Unaware	158	72	45.57	0.624	0.429
		Aware	1,779	869	48.85		
		Total	1,937	941	48.58		
	Senior	Unaware	116	53	45.69	1.585	0.208
		Aware	1,513	783	51.75		
		Total	1,629	836	51.32		
	Postgraduate/doctoral	Unaware	126	61	48.41	15.533	<0.001
		Aware	1,754	1,154	65.79		
		Total	1,880	1,215	64.63		
	All respondents	Unaware	1,045	472	45.17	6.324	0.012
		Aware	11,587	5,704	49.23		
		Total	12,632	6,176	48.89		

Among all respondents, among students who indicated their acceptance of “one-night stands” or online “dates for sex,” the percentage of individuals who possessed AIDS knowledge (6.64, 95% CIs: 6.18–7.09%) was significantly lower than that of individuals who did not possess such knowledge (8.90, 95% CIs: 7.17–10.63%) (χ^2^ = 7.719, *p =* 0.005). In terms of the proportion of students who expressed their disapproval of multiple sexual partners, the percentage of individuals who possessed AIDS knowledge (76.09, 95% CIs: 75.32–76.87%) was significantly higher than that of individuals who did not possess such knowledge (65.84, 95% CIs: 62.96–68.72%) (χ^2^ = 54.136, *p <* 0.001). Among students who reported that they “would use condoms if they had sex,” the percentage of individuals who possessed AIDS knowledge (90.26, 95% CIs: 89.72–90.80%) was significantly higher than that of individuals who did not possess such knowledge (77.03, 95% CIs: 74.48–79.59%) (χ^2^ = 54.136, *p <* 0.001).

### Sexual behaviors

Among all the college students who were interviewed as part of this research, 1,645 (13.02, 95% CIs: 12.44–13.61%) reported that they had engaged in sexual activities, accounting for 14.18% (95% CIs: 13.30–15.07%) of males and 11.98% (95% CIs: 11.20–12.76%) of females.

Among college students who reported engaging in sexual activity, the median age of first sexual activity was 19 years; in particular, 49 students reported that they first engaged in sexual activity at the age of <14 years (2.98%), 201 students reported doing so at an age between 14 and 17 years (12.22%), and 1,395 students reported doing so at 18 years of age or older (84.80%). The proportion of students who reported a history of sexual behavior tended to increase as students’ grade in college increased (χ^2^ trend text = 1411.260, *R* = 0.274, *p* < 0.001); namely, this group of students accounted for 5.16% of freshmen, 11.9% of sophomores, 14.3% of juniors, 15.59% of fourth-year and fifth-year students, 29.27% of master’s students, and 55.22% of doctoral students.

In terms of respondents’ answers concerning their sexual partners, 1,570 students (95.44%) reported sexual relationships with their spouses or heterosexual romantic partners, 104 students (6.32%) reported sexual relationships with ordinary friends, 78 students (4.74%) reported sexual relationships with friends whom they met online, 63 students (3.83%) reported casual sexual relationships on the basis of monetary transactions, and 109 students (6.63, 95% CIs: 5.42–7.83%) reported two or more of the types of sexual partners mentioned above. A total of 109 students (0.86%) indicated that they had been forced to engage in sexual activities.

A total of 1,011 students reported information concerning their sexual partners during the past year; in this context, 807 students (79.82%) reported sexual relationships with their spouses or heterosexual partners, 327 students (32.34%) reported noncommercial sexual activity, 48 (4.75%) reported commercial sexual activities through monetary transactions, and 83 (8.21%) reported being men who had sexual relationships with men.

### Condom use

Among the 1,303 students who reported their frequency of condom use during the past year, 919 (70.53, 95% CIs: 68.05–73.01%) reported that they used condoms each time, 212 (16.27, 95% CIs: 14.26–18.28%) reported that they used condoms occasionally, and 172 (13.20, 95% CIs: 11.36–15.04%) reported that they never used condoms. Among the 48 individuals who reported commercial sex on the basis of monetary transactions, only 9 students (18.75, 95% CIs: 7.30–30.20%) reported that they used condoms each time, 5 students (10.42, 95% CIs: 1.45–19.38%) reported that they used condoms occasionally, and 34 students (70.83, 95% CIs: 57.50–84.17%) reported that they never used condoms. Among the 83 men who had sex with men included in this research, only 40 students (48.19, 95% CIs: 37.22–59.17%) reported that they used condoms each time, 21 (25.30, 95% CIs: 15.75–34.85%) reported that they used condoms occasionally, and 22 (26.51, 95% CIs: 16.81–36.20%) reported that they had never used condoms (see Table 7). During the past year, among respondents who possessed AIDS knowledge, the percentages of students who reported that they used condoms each time they engaged in sexual activities (i.e., 77.58% among those who engaged in such activities with a “spouse or cohabiting heterosexual partner,” 72.16% among those who did so with a “causal sexual partner,” and 48.19% among those who did so with “male same-sex sexual partners”) were greater than the corresponding percentages among students who did not possess AIDS knowledge, and these differences were statistically significant (*p* < 0.01). Students who did not possess AIDS knowledge failed to use condoms each time when they engaged in sexual activities with male same-sex partners (see Table 8).

**Table 7:** Frequency of condom use during sexual intercourse in the last year.

Sexual partner	Every time *n* (%)	Sometimes *n* (%)	Never *n* (%)	χ^2^	*P*
Spouse, cohabiting heterosexualromantic parner^a,b,c^ (*n* = 845)	643 (76.09)	131 (15.50)	71 (8.40)	180.151	<0.001
Temporary sexual partners^a,d,e^ (*n* = 327)	227 (69.42)	55 (16.82)	45 (13.76)		
Commercial sexual partners^b,d,f^ (*n* = 48)	9 (18.75)	5 (10.42)	34 (70.83)		
Same-sex sexual partners^c,e,f^ (*n* = 83)	40 (48.19)	21 (25.30)	22 (26.51)		
Total	919 (70.53)	212 (16.27)	172 (13.20)		

^a,e^ Bonferroni pairwise comparison results, the same letter indicates that the difference is statistically significant (*P* < 0.05). ^b,c,d,f^ Bonferroni pairwise comparison results, the same letter indicates that the difference is statistically significant (*P* < 0.001).

**Table 8:** Comparison between knowledge of HIV/AIDS and frequency of condom use during sexual activity in the past year.

Sexual partner	Knowledge of HIV/AIDS	Every time *n* (%)	Sometimes or never *n* (%)	χ^2^	*P*
spouse, cohabiting heterosexualromantic parner (*n* = 845)	Unaware	41 (59.42)	28 (40.58)	26.231	<0.001
	Aware	602 (77.58)	174 (22.42)		
	Total	643 (76.09)	202 (23.91)		
Temporary sexual partners (*n* = 327)	Unaware	17 (47.22)	19 (52.78)	9.389	0.002
	Aware	210 (72.16)	81 (27.84)		
	Total	227 (69.42)	100 (30.58)		
Commercial sexual partners (*n* = 48)	Unaware	2 (14.29)	12 (85.71)	0.259	0.611
	Aware	7 (20.59)	27 (79.41)		
	Total	9 (18.75)	39 (81.25)		
Same-sex sexual partners (*n* = 83)	Unaware	0 (0.00)	13 (100.00)	12.141	<0.001
	Aware	40 (48.19)	30 (51.81)		
	Total	40 (48.19)	43 (51.81)		

The respondents reported their reasons for failing to use condoms when they engaged in sexual relationships with different partners. The main reasons reported for failing to use condoms during the first sexual act were “thought that there was no need to use” (32.12%), “forgot to use” (16.6%), and “used other contraceptive methods” (1.95%). The main reasons reported for failing to use condoms during sexual activities with one’s spouse or cohabiting heterosexual partners were “I did not think that it was necessary to use it” (41.07%), “I used other contraceptive methods” (17.86%), and “I did not want to use it” (12.50%). The main reasons reported for failing to use condoms during sexual activity with a casual partner were “did not buy condoms” (26.43%), “I did not think that it was necessary to use condoms” (24.53%), “the condoms were too expensive” (9.43%), and “I was embarrassed to buy condoms” (9.43%). The main reasons reported for failing to use condoms during sexual activities with a commercial sex partner on the basis of a monetary transaction were “did not buy condoms” (48.00%), “I did not think that there was any need to use condoms” (16.00%), and “forgot to use condoms” (8.00%). The main reasons reported for failing to use condoms during same-sex sexual activities were “I did not think that it was necessary to use condoms” (41.67%) and “I did not know where to buy condoms” (16.67%) (see Figure 1).

### Preventive services

**Figure 2:**
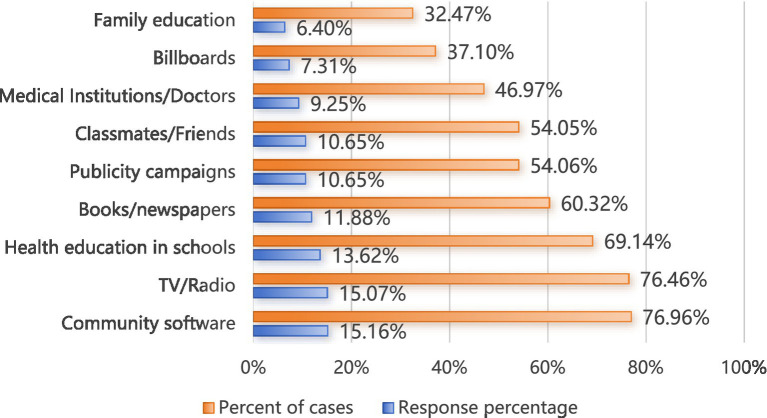
Main sources of HIV/AIDS knowledge (*n* = 12,632).

The college students who were interviewed as part of this research acquired their AIDS knowledge mainly from social software, television/radio, and school health education (see Figure 2).

**Figure 3:**
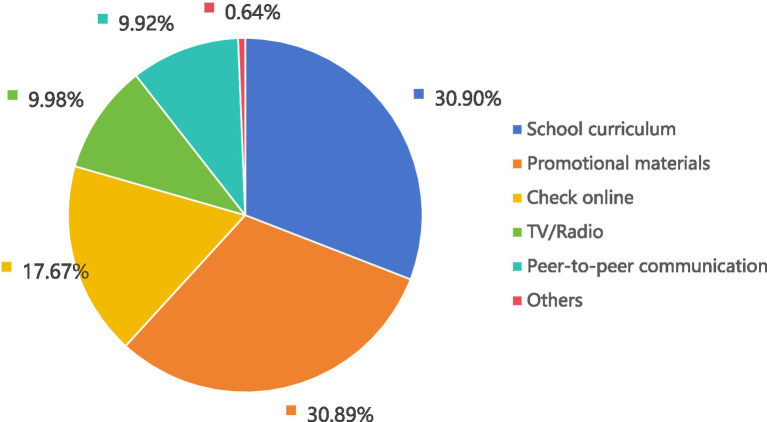
Favorite way to get knowledge about HIV/AIDS (*n* = 12,632).

School courses, publicity materials, and internet inquiries were the most preferable means by which these college students acquired AIDS knowledge (see Figure 3).

**Figure 4:**
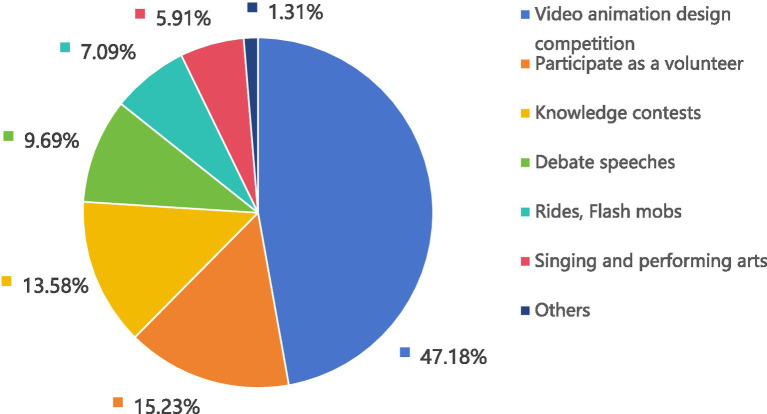
Favorite form of AIDS awareness campaign (*n* = 12,632).

The favorite forms of AIDS publicity activities reported by these college students were video animation design competitions, volunteer participation, and knowledge competitions (see Figure 4).

## Discussion and recommendations

The results of this study revealed that the total level of AIDS prevention knowledge exhibited by the college students who responded to this survey was 91.73%. This percentage was higher than the corresponding figures reported in the following studies: Zeng et al. (11), which focused on medical students in Fujian Province in southeastern China from 2022 to 2023 (72.41%); Zhang et al. (12), which focused on college students in Henan Province in central China in 2020 (80.8%); Hu et al. (13), which focused on college students recruited from 5 colleges and universities in Hunan Province in central-southern China from 2022 to 2023 (86.34%); Tu et al. (14), which focused on junior college students in Jiangsu Province in central and eastern China (87.4%); and Liu et al. (15), which focused on junior college students in Tianjin (87.33%) in northern China. The differences in the results of these surveys, which were conducted in different regions of China, may be related to the lack of unified policies and teaching standards pertaining to the health education of college students across different regions. Only 44.85% of the respondents answered all eight questions correctly, which revealed a tremendous gap with respect to the goal of “China’s AIDS Containment and Prevention Program (2024–2030)” (16), which uses “more than 95% awareness among key populations and groups that engage in HIV risk behaviors” as a working indicator. Only 2 items were associated with awareness rates higher than 95% (i.e., the awareness rate of q5 was 95.74%, and the awareness rate of q7 was 97.97%), and the awareness rates associated with the remaining 6 items ranged between 80.84 and 88.30% (in particular, q2, “The current prevalence of HIV among young students in China is rapidly increasing, and the main mode of transmission is male homosexual behavior, followed by heterosexual sexual behavior,” was associated with the lowest awareness rate of 80.84%, while the awareness rate of q1, “AIDS is a serious incurable infectious disease,” was 81.86%). These results were similar to those that have been reported by various foreign scholars who have conducted similar surveys (17–19). The lack of a correct understanding of the incurable nature of AIDS seriously affected the level of importance that college students attach to HIV prevention. Ignorance of sexual behavior, which is the main route of transmission, also affected perceptions of risky sexual behavior among college students and the implementation of more effective measures in this context. Regarding personal protection measures, Chinese universities should strengthen their methods of AIDS prevention and control with the aim of spreading AIDS knowledge among college students and raising awareness of the need for self-protection in this regard.

The results of this study revealed that there were no significant differences in the levels of AIDS knowledge among college students of different genders. This finding was similar to those of Qashqari et al. (20), who surveyed 2,081 residents of the Kingdom of Saudi Arabia, Thanduxolo (21) who surveyed 422 students aged 15–21 years in South Africa, and Dzah et al. (18), who surveyed adolescents aged 15–24 years in Ghana. Khan et al. (22) conducted a survey of the general population in Kyrgyzstan; the results revealed that females were significantly higher than males in terms of correct knowledge of mother-to-child transmission and blood-borne transmission, but there were no significant differences in knowledge of sexual transmission. The differences in the findings of different scholars may be related to the level of local national education and the strategies used to promote AIDS knowledge.

The results of this study revealed that college students who were 20 years old or older exhibited higher levels of AIDS knowledge. Furthermore, the level of AIDS prevention knowledge exhibited by college students in higher grades was higher than that students in lower grades, and the level of such knowledge exhibited by graduate students was higher than that exhibited by undergraduate students. Moreover, college students acquired AIDS knowledge mainly from social software, television/radio, and school health education; this finding may be related to the characteristics of Chinese college students and the educational methods used in China. Most high schools in China do not allow students to bring mobile phones to school. Parents also limit the time available to high school students to surf the internet or watch television. In addition, Chinese high schools rarely provide courses in AIDS health education. The junior college students who participated in this study, especially freshmen who enrolled in September, had been enrolled for only 2 months at the time of the survey. Therefore, with the exception of some universities that offered AIDS health education courses after enrolment, the level of knowledge exhibited by these freshman was equivalent to that of high school students. Liu et al. (23) conducted a study in Qinghai Province, China; the results revealed that the level of AIDS knowledge among freshmen who received school health education increased from 48.59% before they received such education to 76.24% thereafter. School health education is an important strategy that can be used to improve college students’ AIDS knowledge (14, 24). Previous studies have reported that junior college students who seek sexual freedom face the risk of HIV transmission and that providing HIV health education as soon as possible is a protective factor with respect to college students’ sexual behavior (25–27). Shokoohi et al. (28) divided Iranian youth aged 15–29 into three age groups: 15–18 years old, 19–24 years old, and 25–29 years old; the results revealed that the level of AIDS knowledge among young people in the upper age group was significantly higher than that in the lower age group. Khargekar et al. (29) conducted a survey of 401 undergraduate and graduate students in four universities and colleges in India and found that age, gender, and education level had no significant impact on AIDS knowledge. The discrepancies in these findings further suggest that it is important for governments to conduct adequate surveys in the areas where the policies are implemented and to formulate more suitable prevention and control strategies based on the actual conditions in their regions.

The results of a multivariate logistic regression analysis revealed that the level of AIDS knowledge exhibited by students in mainland China was 2.5 times that exhibited by students in Hong Kong, Macao, and Taiwan. This finding may be related to differences in the distribution of AIDS health education resources across schools that operate on the basis of different systems in China (30, 31). Colleges and universities in mainland China should thus focus on the students from Hong Kong, Macao and Taiwan with respect to the development of AIDS health education. The level of AIDS knowledge exhibited by college students whose average monthly living expenses were RMB 1,000 or higher was nearly 1.7 times that exhibited by other college students. This finding may be related to the fact that students whose monthly living expenses are low pay less attention to knowledge pertaining to AIDS prevention and control (32).

The results of this study suggested that the proportion of students who reported a history of sexual behavior increased alongside their grade in college; namely, the percentages of sophomores (11.9%), fourth-year students and fifth-year students (15.59%), master’s students (29.27%), and doctoral students (55.22%) were approximately one, three, six and 11 times that of the freshmen (5.16%), respectively. The overall rate of sexual behaviors among college students identified in this survey was 13.02%, which was higher than the value reported by Liu et al. (33) on the basis of a convenience sampling survey of 7,346 students across 5 universities in Guangzhou, which was conducted between November and December 2021 (9.08%). The overall rate of sexual behaviors was lower than has been reported in a meta-analysis of the rate of sexual behavior among college students in mainland China (15.1%) (34); this difference may be related to the relatively high proportion of junior students included in this survey. A total of 6.63% of the respondents who reported engaging in sexual intercourse mentioned that they had noncommercial sex with casual partners; this value was lower than a corresponding figure that has been reported in Zhejiang, China (20%) (35). A total of 3.83% of the respondents who reported engaging in sexual activities mentioned that they had casual sexual relations on the basis of monetary transactions; this value was lower than a corresponding figure that has been reported in Beijing, China (17.7%) (36).

In the past year, the percentages of students who reported that they insisted on using condoms when they engaged in sexual activities with heterosexual partners (76.09%) and casual sexual partners (69.42%) were higher than the corresponding figures reported by Chen et al. (37), who surveyed college students from three universities in Shenzhen between October 2015 and January 2016 (heterosexual couples 61.72%, casual sexual partners 55.22%). However, the percentage of students who reported that they insisted on using condoms during sexual activities was lower than of the corresponding figures reported by Chen et al. (37) with regard to individuals who engaged in sexual activities with commercial sexual partners (18.75% vs. 79.79%) or same-sex sexual partners (48.19% vs., 56.52%). The percentage of students who reported that they insisted on using condoms when they engaged in sexual activities with casual partners during the past year was also higher than the corresponding figure (40.09%) reported by Yan et al. (38), who surveyed college students at 13 universities in Zhejiang Province in 2018. The percentages of students who possessed AIDS knowledge and used condoms each time they engaged in sexual activities with “casual sexual partners” or “male same-sex partners” during the past year were greater than the corresponding percentages of students who did not possess AIDS knowledge, thus indicating that increasing AIDS knowledge is helpful with respect to efforts to promote condom use. Individuals who did not possess AIDS knowledge reported that they did not use condoms each time they engaged in sexual activities with male same-sex partners, thus increasing the risk of HIV transmission via male homosexual sex among college students. The main reason for failing to use condoms during sexual activities with casual sexual partners (26.43%) and commercial sexual partners on the basis of monetary transactions (48.00%) was “did not buy condoms.” The main reason for failing to use a condom during same-sex sexual activity was “I did not think that it was necessary to use condoms” (41.67%). These findings may be related to the insufficient risk assessment ability of college students with respect to sexual behavior. The promotion and use of condoms are important components of interventions targeting high-risk AIDS behavior (39). The task of enabling college students to assess the risks entailed by sexual behavior correctly and to employ appropriate coping methods should be a major focus of future research on this topic.

The proportion of the college students included in this survey who reported that they had acquired AIDS knowledge from social software was the highest (76.96%); this finding was similar to the results reported by Ajwah et al. (40). However, nearly one-third of the respondents reported that their favorite means of acquiring AIDS knowledge was school courses (30.90%), and only 17.67% reported that their favorite means of acquiring such knowledge was the internet; these findings were consistent with the observations of Deng et al. (41) in Huizhou, China, However, such findings were not consistent with the observations of Tsegay et al. (42), who reported that most students prefer to acquire AIDS knowledge via the internet. This study reveals that students typically hope to acquire AIDS knowledge from schools; however, schools have failed to meet the needs of students, thus leading students to turn to the internet as a source of information. Mastery of correct AIDS knowledge is an important foundation for efforts to reduce high-risk behaviors as well as the risk of HIV transmission (43). AIDS knowledge courses can be developed in schools by combining traditional onsite teaching with online classrooms, social platforms and organized activities with the goals of meeting the knowledge needs of college students and enhancing their AIDS knowledge across multiple dimensions (44, 45).

This study shows that the overall level of AIDS knowledge among college students in Guangzhou, China, was relatively high, but the correct understanding of some of the knowledge was still insufficient. The level of AIDS knowledge of college students was affected by a variety of factors, and policymakers should provide multi-level interventions for students in the lower grades, non-Chinese mainland students (Hong Kong, Macao, and Taiwan students), students living in student dormitories, sexual history, and low average monthly living expenses, so as to further improve the level of AIDS knowledge of college students. The AIDS knowledge level of college students was affected by a variety of factors, and policymakers should provide multi—level interventions for lower—grade students, non—Chinese mainland students (Hong Kong, Macao, and Taiwan students), students living in dormitories, students with sexual history, and students with low average monthly living expenses, so as to further improve the AIDS knowledge level of college students.

## Data Availability

The datasets presented in this study can be found in online repositories. The names of the repository/repositories and accession number(s) can be found in the article/supplementary material.

## Footnotes

^1^ https://www.wjx.cn

## References

[1] Money DM. HIV/AIDS is not over. J Obstetr Gynaecol Canada. (2022) 44:1240–1. doi: 10.1016/j.jogc.2022.10.007 36567089

[2] Thornton J. Botswana's HIV/AIDS success. Lancet. (2022) 400:480–1. doi: 10.1016/S0140-6736(22)01523-9 35964598

[3] UNAIDS. Latest global and regional HIV statistics—fact sheet 2024. (2024).

[4] WHO. World health statistics 2024: Monitoring health for the SDGs, sustainable development goals. Geneva: WHO (2024).

[5] UNICEF. Adolescent HIV prevention: In order to ramp up our efforts in the fight against AIDS, there is a need for more concentrated focus on adolescents and young people. (2024).

[6] UNAIDS. HIV/AIDS JUNPo: The path that ends AIDS: UNAIDS global AIDS update 2023. Geneva: UNAIDS (2023).

[7] He N. Research progress in the epidemiology of HIV/AIDS in China. China CDC Weekly. (2021) 3:1022–30. doi: 10.46234/ccdcw2021.249, PMID: 34888119 PMC8633551

[8] Han MJ Chen QF Xu P Shi Y. Strive for a new journey in AIDS prevention and control during the 13^th^ five-year: review and prospect of AID Sprevention and control in China. Chin J AIDS STD. (2021) 27:1327–31. doi: 10.13419/j.cnki.aids.2021.12.01

[9] Cai C Tang HL Chen FF Li DM Lyu P. Characteristies and trends of newly reported HIV infecting in young students in China, 2010-2019. Chin J Epidemiol. (2020) 41:1455–9. doi: 10.3760/cma.j.cn112338-20200417-00592 33076598

[10] National Center for AIDS/STD Control and Prevention, China CDC. Questionnaire on HIV/AIDS awareness among young students. Available online at: https://www.chinaaids.cn/qsnazbfk/xzzx/201705/P020170510598644914191.pdf.

[11] Zeng YH Kong XR Cao WN Shi ZL Shi YH Zheng YT. Current situation of AIDS knowledge among students in a medical college in Fujian Province and analysis of relevant policies in local area. Chinese J Health Educ. (2024) 11:1038–46. doi: 10.16168/j.cnki.issn.1002-9982.2024.11.015

[12] Zhang L Yu H Luo H Rong WL Meng XX Du XA. HIV/AIDS-related knowledge and attitudes among chinese college students and associated factors: across-sectional study. Front Public Health. (2022) 9:9. doi: 10.3389/fpubh.2021.804626, PMID: 35096751 PMC8790097

[13] Hu M Zou XB He JM Zheng J Chen X. Awareness rate of knowledge about AIDS and sexual behavior among university students in Hunan Province, 2022-2023. Pract Prev Med. (2024) 31:1310–3. doi: 10.3969/j.issn.1006-3110.2024.11.007

[14] Tu FL Yang RZ Li R Du GP LYY Li W Wei PM. Structural equation model analysis of HIV/AIDS knowledge, attitude, and sex education among freshmen in Jiangsu, China. Front Public Health. (2022) 10:10. doi: 10.3389/fpubh.2022.892422, PMID: 35664113 PMC9159914

[15] Yi L Liu ZQ Wu ZM Gong H Bai JY Yu MH. Current status of AIDS knowledge, attitudes, practices and associated factors of high-risk sexual behavior among college students in Tianjin City. Chin J School Health. (2024) 45:203–206, 212. doi: 10.16835/j.cnki.1000-9817.2024053

[16] General Office of the State Council of the People's Republic of China. Circular of the General Office of the State Council on Printing and Distributing China's Plan for Containing and Combating HIV/AIDS(2024-2030). Chinese J AIDS STD. (2024) 30:1227–9.

[17] Avina RM Mullen M Mshigini S Becerra MB. I actually don’t know what HIV is: a mixed methods analysis of college students’ HIV literacy. Diseases. (2020) 8:1. doi: 10.3390/diseases8010001, PMID: 31906556 PMC7151332

[18] Dzah SM Tarkang EE Lutala PM. Knowledge, attitudes and practices regarding HIV/AIDS among senior high school students in Sekondi-Takoradi metropolis, Ghana. Afr J Prim Health Care Family Med. (2019) 11:e1–e11. doi: 10.4102/phcfm.v11i1.1875 PMC6556927 31170791

[19] Orlando G Campaniello M Iatosti S Grsdale PJ. Impact of training conferences on high-school students’ knowledge of sexually transmitted infections (STIs). J Prev Med Hyg. (2019) 60:E76–83. doi: 10.15167/2421-4248/jpmh2019.60.2.1072, PMID: 31312736 PMC6614570

[20] Qashqari FS Alsafi RT Kabrah SM AlGary RA Naeem SA Alsulami MS. Knowledge of HIV/AIDS transmission modes and attitudes toward HIV/AIDS infected people and the level of HIV/AIDS awareness among the general population in the Kingdom of Saudi Arabia: a cross-sectional study. Front Public Health. (2022) 10:955458. doi: 10.3389/fpubh.2022.955458, PMID: 36238245 PMC9551601

[21] Thanduxolo F. Knowledge, attitude and practices regarding HIV and aids among high school learners in South Africa. Open AIDS J. (2021) 15:84–92. doi: 10.2174/1874613602115010084

[22] Khan NH Beg M Sarwar MZ Kyzy GZ Zhetkinbekova T Mamatov A. Assessment of knowledge and attitudes related to HIV/AIDS among the population with increasing incidence rate. Cureus. (2024) 16:e53451. doi: 10.7759/cureus.53451, PMID: 38435229 PMC10909385

[23] Liu Y Lu L Wang YY Meredith RW Ren YM Wang CC. GAO J, LIU S: effects of health education on HIV/AIDS related knowledge among first year university students in China. Afr Health Sci. (2020) 20:1582–90. doi: 10.4314/ahs.v20i4.10, PMID: 34394218 PMC8351845

[24] Nutbeam D. From health education to digital health literacy - building on the past to shape the future. Glob Health Promot. (2021) 28:51–5. doi: 10.1177/17579759211044079, PMID: 34719292

[25] Zou HC Tucker JD Fan S Xu JJ Yu MH Luo ZZ. Learning about HIV the hard way: HIV among Chinese MSM attending university. Lancet Infect Dis. (2018) 18:16–8. doi: 10.1016/S1473-3099(17)30711-9, PMID: 29303730

[26] Duncan C Miller DM Borskey EJ Fomby B Dawson P Davis L. Barriers to safer sex practices among African American college students. J Natl Med Assoc. (2002) 94:944–51. PMID: 12442997 PMC2594191

[27] Zhang DG Pan H Cui BL Law F Farrar J William BT. Sexual behaviors and awareness of sexually transmitted infections among Chinese university students. J Infect Dev Ctries. (2013) 7:966–74. doi: 10.3855/jidc.3872, PMID: 24334944

[28] Shokoohi M Karamouzian M Mirzazadeh A Haghdoost A Rafierad AA Sedaghat A. HIV knowledge, attitudes, and practices of young people in Iran: findings of a national population-based survey in 2013. PLoS One. (2016) 11:e0161849. doi: 10.1371/journal.pone.0161849, PMID: 27626638 PMC5023173

[29] Khargekar N Takke A Athalye S Panale P Rajamani N Banerjee A. Exploring factors influencing the perspective regarding HIV transmission and prevention among college students in India. J Family Med Prim Care. (2024) 13:1467–72. doi: 10.4103/jfmpc.jfmpc_1756_23, PMID: 38827717 PMC11141968

[30] Zhu Z Guo M Petrovsky DV Dong T Hu Y Wu B. Age and regional disparity in HIV education among migrants in China: migrants population dynamic monitoring survey, 2014-2015. Int J Equity Health. (2019) 18:104. doi: 10.1186/s12939-019-0999-x 31269954 PMC6610800

[31] Zhang R Chen L Cui YD Li G. Achievement of interventions on HIV infection prevention among migrants in China: a meta-analysis. SAHARA J. (2018) 15:31–41. doi: 10.1080/17290376.2018.1451773, PMID: 29564968 PMC5917330

[32] Li HL Wu Q Gao EZ Zhang Y Yin DH. HIV/AIDS-related knowledge and attitudes toward people living with HIV among college students in Xuzhou, Jiangsu Province, China: a cross-sectional survey. Front Public Health. (2024) 12:1398980. doi: 10.3389/fpubh.2024.1398980, PMID: 39450388 PMC11500070

[33] Liu J Lin P Xu HF Yang F Fu XB Yao ZL. High-risk sexual behaviors of HIV/AIDS and related factors in young students in Guangzhou. Chinese J Epidemiol. (2024) 45:265–72. doi: 10.3760/CMA.J.CN112338-20230617-00383 38413067

[34] Yang YM Shen YL Li SY. Occurrence of sexual behavior among college students in mainland China: a meta-analysis. Chin J Public Health. (2018) 34:142–7.

[35] Yang ZR Chen WY Jin MH Chen WJ Chen L Zhou X. Analysis of factors influencing casual sexual behavior among male college students in Zhejiang province, China. PLoS One. (2021) 16:e0250703. doi: 10.1371/journal.pone.0250703, PMID: 33939731 PMC8092760

[36] Cui JC Zhang X Chang C Sun XY Fan XY Shi YH. A comparative study on safe sexual behavior and its intention among college students in Beijing between 2006 and 2016. Chinese J Dis Control Prev. (2019) 23:1191–5. doi: 10.16462/j.cnki.zhjbkz.2019.10.007

[37] Chen WY Ma QQ Zhou X Chen WJ Jiang TT Wang H. Analysis on condom use in 424 male college students with casual heterosexual behaviors in Zhejiang. Dis Surv. (2022) 37:492–7. doi: 10.3784/jbjc.202107230414

[38] Yan Y Yan M Luan RS Zhang XX Xu SK. Study on the sexual behavior and its influencing factors among college students in Shenzhen, Guangdong. Chin J Sch Health. (2016) 37:1784–6. doi: 10.16835/j.cnki.1000-9817

[39] Su XY Zhou AN Li JJ Shi LE Huan XP Yan HJ. Depression, loneliness, and sexual risk-taking among HIV-negative/unknown men who have sex with men in China. Arch Sex Behav. (2018) 47:1959–68. doi: 10.1007/s10508-017-1061-y, PMID: 29147806 PMC5955768

[40] Ajwah IM Alamri SA Alatawi AS Alhawiti RM Mirghani HO. A study of the knowledge about HIV disease (AIDS) among literary section students in Tabuk University. Basic Res J Med Clin Sci. (2017) 6:20–3.

[41] Deng WF Huang L Qiu WQ. Investigation on knowledge awareness rate and prevention needsof AIDS among 12 109 college students in Huizhou City 2021. J Prev Med Informat. (2024). doi: 10.19971/j.cnki.1006-4028.240528

[42] Tsegay G Edris M Meseret S. Assessment of voluntary counseling and testing service utilization and associated factors among Debre Markos university students, north West Ethiopia: a cross-sectional survey in 2011. BMC Public Health. (2013) 13:243. doi: 10.1186/1471-2458-13-243, PMID: 23509888 PMC3641945

[43] Groenendijk AL Vos WAJW Dos Santos JC Rokx C van der Ven AJAM Verbon A. Non-AIDS events in individuals with spontaneous control of HIV-1:a systematic review. J Acquir Immun Defic Syndr. (2022) 91:242–50. doi: 10.1097/QAI.0000000000003066, PMID: 35969465

[44] Zheng YT Zhang X Sun XY Shi YH Chang C. Evaluation of the college-based HIV/AIDS education policy in Beijing, China: a mixed method approach. Environ Health Prev Med. (2020) 25:50. doi: 10.1186/s12199-020-00890-5, PMID: 32912181 PMC7488098

[45] Jones SG Chadwell K Olafson E Simon S Fenkl E Framil CV. Effectiveness of nursing student-led HIV prevention education for minority college students: the SALSA project. J Health Care Poor Underserved. (2017) 28:33–47. doi: 10.1353/hpu.2017.0051, PMID: 28458263

